# Traumatic Thoracic Blast Injury With Extensive Soft Tissue Damage, Volumetric Muscle Loss, and Full-Thickness Burns From a Flashbang Detonation: A Case Report

**DOI:** 10.7759/cureus.74920

**Published:** 2024-12-01

**Authors:** Sahil Patel, Roberto Echeverri

**Affiliations:** 1 Medicine, Dr. Kiran C. Patel College of Osteopathic Medicine, Nova Southeastern University, Fort Lauderdale, USA; 2 Department of Family Medicine, Miami Veterans Affairs Medical Center, Miami, USA

**Keywords:** advancement flap, blast injury, burn wound care, chest wall trauma, muscle loss, primary surgical repair, soft tissue damage

## Abstract

Chest trauma is a common injury in civilian and military polytrauma patients and is a leading cause of death and chronic disabilities. Evolving surgical techniques and advancements in chest wall reconstruction have improved clinical outcomes, reduced operative complications, and decreased mortality. In this case report, a 23-year-old male U.S. Army soldier sustained a left chest wall and shoulder blast injury with significant soft tissue damage, volumetric pectoralis major muscle loss, mild sensory nerve damage, and third-degree burns from a flashbang grenade. The initial treatment involved wound debridement, IV antibiotics, and pain management. This was followed by surgical reconstruction using a local advancement flap to prevent infection, address tissue loss, and restore chest wall integrity. Postoperative care included pain management and physical therapy, leading to a successful recovery with restored mobility and chest wall stability. This case highlights the challenges of treating severe chest wall trauma with burns, emphasizing the importance of timely surgical intervention and a multidisciplinary approach.

## Introduction

Severe accidental blast injuries during military training are an uncommon pathology, but when coupled with third-degree burns following a flashbang grenade detonation, the case becomes particularly unique. In patients with polytrauma, chest trauma is the second most common unintentional traumatic injury and the third most common cause of death after abdominal and head trauma [1]. Immediate surgery has been cited as the best treatment for pectoralis major muscle injuries, with these patients most likely to result in a return to full-level activity [2]. With recent medical advancements, there has been a marked improvement in the management and reconstruction of complex chest wall defects. Through the utilization of muscle and musculocutaneous flaps, such as the latissimus dorsi, pectoralis major, serratus anterior, and rectus abdominis, there has been a decrease in infections and mortality [3]. In this case, the patient required surgical reconstruction of the chest wall to obliterate dead space, restore chest wall rigidity, preserve pulmonary mechanics, protect intrathoracic organs, provide soft tissue coverage, and minimize deformity [4]. Full-thickness defects of the chest wall may be surgically repaired in a single procedure, with the pectoralis major being the principal flap for sternal and anterosuperior chest wall defect coverage [5]. When the resection has not compromised skeletal stability, and if vital structures are not exposed, a combination of skin grafts, local skin flaps, and negative-pressure wound therapy may be the most advantageous procedure [6]. Postoperative rehabilitation is critical to achieving a positive outcome as a high body mass index and active psychiatric diagnoses are major risk factors in postoperative failure and the inability to regain function [7].

## Case presentation

Patient history and appearance

A 23-year-old male U.S. Army enlisted soldier was injured during an advanced military building clearance tactics training. Upon entering a room with fellow soldier trainees, a flashbang grenade was thrown inside, rebounded off the wall, and detonated near his chest wall and left shoulder. Military medical personnel at the scene of the incident immediately acted by packing the wound with dressings to control the bleeding, and an 18-gauge IV line with normal saline was established in the left antecubital area. The patient was subsequently taken via ambulance to the nearest emergency department. The patient was observed throughout transport to the emergency department and was hemodynamically stable.

Upon arrival at the emergency department, the patient presented as conscious and oriented to person, place, and time (COAx3). Vital signs at arrival were as follows: blood pressure 129/64 mmHg, pulse 64 beats per minute, respiratory rate 16 breaths per minute, O_2_ saturation 100%, and temperature 98.8°F (Table 1). Pupils were equal, round, and reactive to light (PERRL). The respiratory rate was regular, and there were no signs of distress or labored breathing. Pulses were regular and strong. The cardiac monitor showed normal sinus rhythm (NSR) with a rate of 67 bpm. Blood glucose was measured at 129+ mg/dL. The wound was localized over the left chest and shoulder, measuring 16 cm x 12 cm x 3 cm with irregular borders. The wound exposed muscle, and the surrounding area showed full-thickness burns covering 2% of the body surface area. Intravenous (IV) cefazolin 2 grams was administered. The patient reported pain, which was managed with intravenous (IV) morphine 10 milligrams and oral analgesics. No motor deficits but mild sensory deficits were observed in the upper extremity. Wet gauze was applied to the wound, which was redressed as needed due to persistent bleeding. The patient, in stable condition, was subsequently moved to a trauma and burn care center for specialized treatment.

**Table 1:** Lab results ordered during hospital admission. Note: All undocumented lab findings were in the normal range. WBC: white blood cell; RBC: red blood cell; ABS: absolute; INR: international normalized ratio

Lab test	Specimen source	Result	Reference range
WBC	Blood	11.3 x 10(3)/mcL	4.9-10.0 x 10(3)/mcL
RBC	Blood	4.56 x 10(6)	4.7-6.1 x 10(6)
Hemoglobin	Blood	13.0 g/dL	14.0-18.0 g/dL
Hematocrit	Blood	38.6%	42%-52%
ABS Neutrophils	Blood	8.1 x 10(3)/mcL	1.4-6.5 x 10(3)/mcL
Neutrophils	Blood	71.5%	30%-70%
Lymphocytes	Blood	18.1%	20%-40%
Protime	Plasma	15.1 sec	10.7-13.9 sec
INR	Plasma	1.29	2-3

Lab findings

Imaging

**Figure 1:**
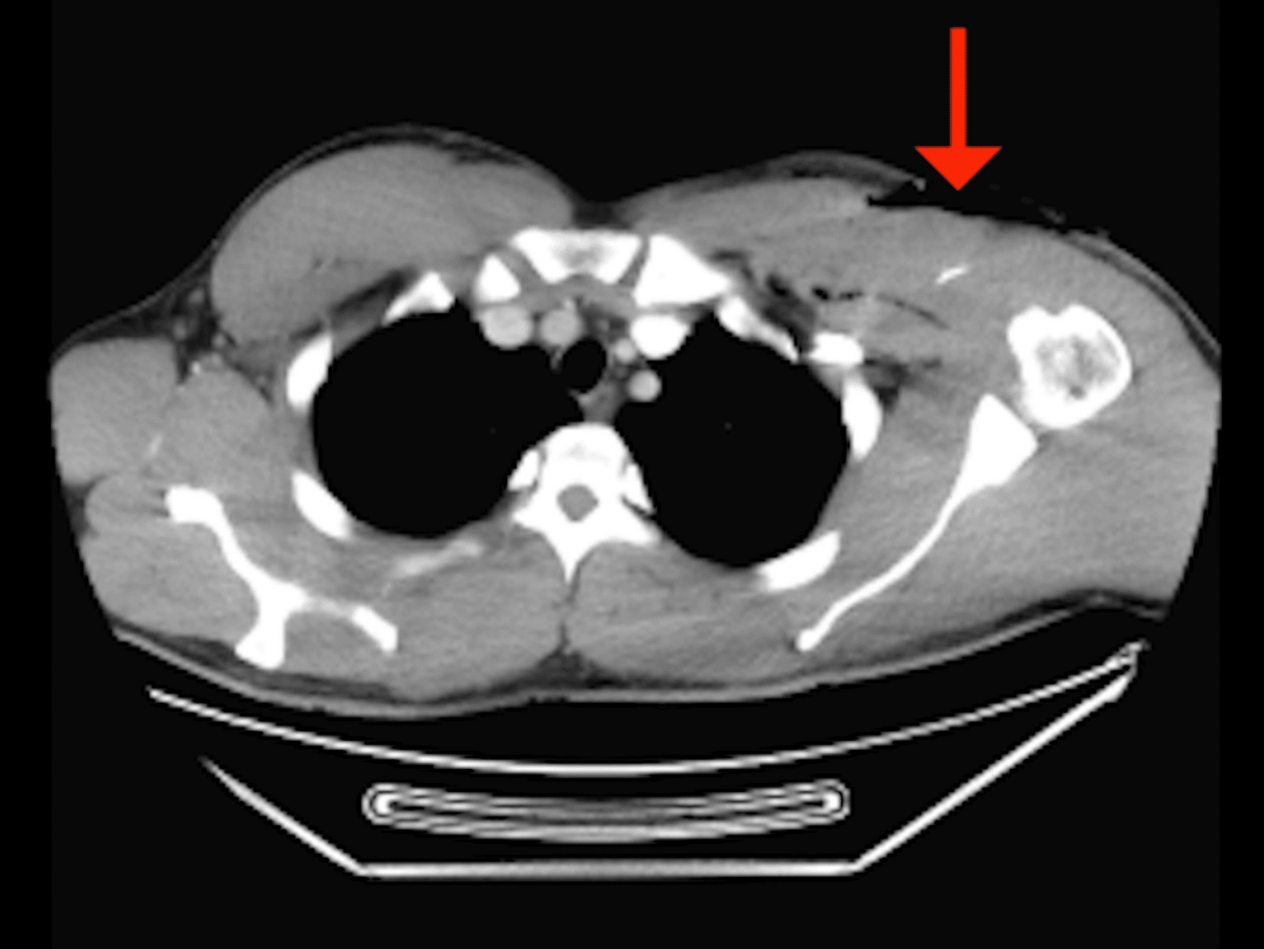
Chest CT showed soft tissue disruption on the left anterior upper chest wall, accompanied by a significant skin defect, muscle loss, and subcutaneous emphysema surrounding the left pectoralis major.

X-ray with an AP view of the chest showed adequate lung volumes, no focal consolidations, clear costophrenic angles, no pneumothorax, cardiac silhouette within normal limits, unremarkable soft tissues and osseous structures, and no acute cardiopulmonary disease. A contrast-enhanced neck CT showed normal findings, with no significant abnormalities or foreign bodies. Axial 2.5 mm CT images were obtained through the thorax (Figure 1), showing soft tissue disruption over the left anterior superior chest wall with a large skin defect, subcutaneous emphysema extending around the left pectoralis major, and no evidence of pneumothorax, underlying fracture or dislocation, or apparent vascular injury.

Surgical course

The patient underwent his first surgical intervention one day following the accident for wound debridement and washout. The wound was classified as a Class III contaminated open wound with necrotic tissue and foreign bodies. Two grams of IV cefazolin was given as a prophylactic antibiotic. The surgery began with a sharp incision into the skin edges of the wound with a 10-blade scalpel. Inspection of the wound bed revealed necrotic subcutaneous fat and muscle, which was incised down to viable tissue that contracted when electrocautery was applied to it. The wound bed was then washed with five liters of normal saline. Surgical findings included full-thickness debridement of 25 cm² of burned skin, removal of 125 cm² of necrotic fat and muscle, and extraction of three small plastic fragments from the flashbang casing. The wound measured 16 cm x 12 cm x 3 cm and was packed with gauze soaked in normal saline. Dry gauze was applied on top of this, and a mesh dressing was placed to hold the dressing in place. IV cefazolin was continued to prevent infection.

Six days after the injury, a second washout and debridement were performed due to persistent contamination and remaining necrotic tissue. The surgical team extended the incision both longitudinally toward the chest and shoulder, removing more debris, carbonized and necrotic muscle, and skin. In addition, a small plastic foreign body was removed from the left shoulder. The pectoralis muscle was split but was not deep into the patient's shoulder joint. Further necrotic tissue measuring 10 cm² was excised. The wound was irrigated copiously with six liters of normal saline with the Simpulse irrigation. Hemostasis was obtained using Bovie electrocautery. The wound measured 16 cm x 6 cm, was packed with gauze soaked in quarter-strength Dakin solution, and covered with Tefla Clear and Gentamicin. Strips of Ace bandage were placed around the periphery of the wound and pulled over the burn fluff creating a compressive dressing for this wound. The patient tolerated the procedure well and was prepared for a subsequent closure procedure. On the 12th day of inpatient care, the patient was hemodynamically stable and underwent definitive wound closure through a local advancement flap using skin and tissue from the immediate area around the chest wall defect. An Allis retractor and rakes were used to secure the lower body flap at the superior and inferior aspects of the wound. The flap was then elevated with the Allis retractor by carefully separating the subcutaneous tissue from the underlying muscle fascia. The dissection was extended to the area just above the nipple, allowing the flap to cover the entire chest region. The surgical flap was elevated in multiple directions using electrocautery to ensure precision. Medially, the dissection extended toward the sternum, carefully raising the tissue along its course. Laterally, the flap was advanced toward the axilla, maintaining a clear separation between the fat and the underlying fascia. Superiorly, the dissection continued upward, extending beyond the shoulder and progressing nearly to the back.

**Figure 2:**
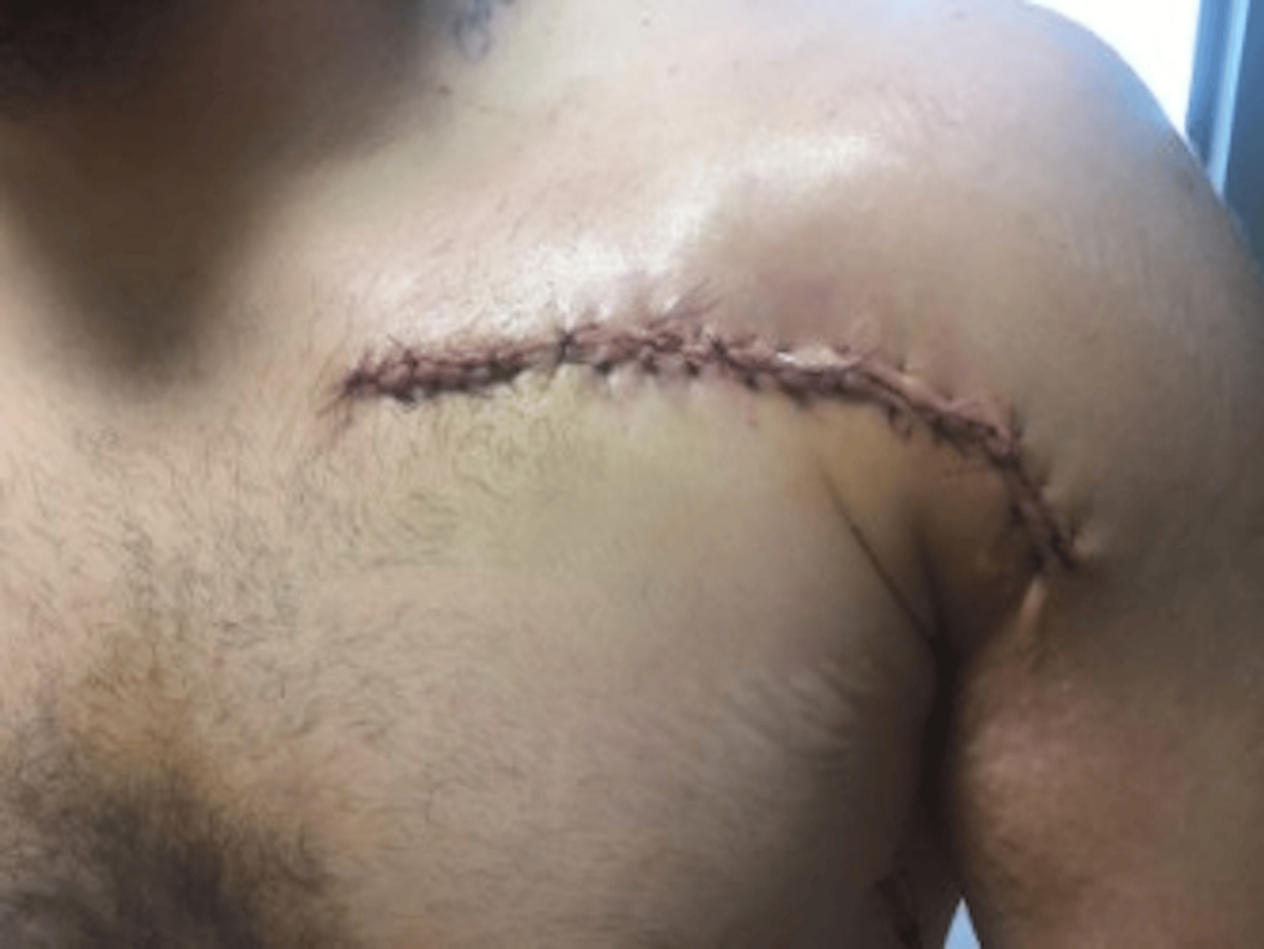
Deep sutures were placed with 2-0 Vicryl, and the superficial layers were closed using interrupted running vertical mattress sutures with 4-0 Monocryl. The closure was complex, with the final wound measuring approximately 60 cm in length and closed with moderate tension.

This approach ensured adequate flap mobility while preserving the integrity of the surrounding structures. The fasciocutaneous flaps were then mobilized superiorly, inferiorly, medially, and laterally. The skin, subcutaneous tissue, and underlying fascia were carefully approximated and secured to close the space of the wound. The wound, now measuring 16 cm x 12 cm, was irrigated again with the Simpulse, and hemostasis was maintained using electrocautery. A 10-mm Jackson-Pratt drain was placed under the flaps via a separate stab incision in the left axilla and secured with 3-0 nylon sutures. Once hemostasis was confirmed, the wound was closed in layers. The deep sutures were 2-0 Vicryl and the superficial sutures were interrupted by running vertical mattress sutures with 4-0 Monocryl (Figure 2). The wound was about 20 cm long upon closing the entire wound with a reasonable amount of tension. For postoperative pain control, 20 mL of 0.25% plain Marcaine was injected around the wound periphery. A dressing was applied, and the patient tolerated the procedure well. The patient was extubated in the operating room and transferred to the recovery room in stable condition, with plans for admission to the burn unit for postoperative care.

Postoperative care and recovery

Following wound closure, the patient remained hemodynamically stable, with no signs of infection at the surgical site and stable vital signs. The Jackson-Pratt drain output was closely monitored and removed five days following the procedure, after two consecutive days of less than 20 cc of output. Physical and occupational therapy was initiated on the fourth postoperative day to maintain shoulder mobility and prevent scar adhesion. The patient was completing activities of daily living independently but experienced left shoulder and chest pain with a limited range of motion. He had an AM-PAC Score (Activity Measure Post-Acute Care Score) of 23/24, indicating up to a 19% level of impairment. The patient was admitted for 17 days, and at the time of discharge, he was afebrile, had stable vitals, tolerated a regular diet, and ambulated without assistance. His home medication instructions included bacitracin ointment to be applied daily, 800 mg ibuprofen every six hours for pain, and oxycodone acetaminophen 325 mg every six hours for fix days for pain. He was provided with a detailed postoperative care and recovery plan for continued rehabilitation and wound care. Physical therapy placed the patient in a home exercise program for the left shoulder. A follow-up visit with surgery two weeks postop confirmed that the wound was healing appropriately with no postoperative complications. A dermatology visit four weeks postop noted a linear erythematous thick scar with minimal scar spread centrally that should revert to normal in six months.

## Discussion

Significant chest wall trauma is a complex pathology that poses a considerable challenge to hospitalists and surgeons. When caused by a flashbang explosion, it becomes a rare occurrence requiring a unique approach to management due to its potential for complications. This type of injury often presents significant diagnostic challenges, necessitating both perioperative care and surgical intervention. However, a timely diagnosis and prompt intervention can yield excellent patient outcomes and therapeutic relief. Trauma of the chest is associated with high mortality; therefore, an adequate primary survey, understanding of the underlying injury, and early intervention are key factors for survival after trauma [8].

In our case, the patient presented to the emergency department shortly after the injury, and immediate evaluation with imaging allowed for a proper understanding and precise location of impact on the chest wall. The injury was located over the left pectoralis major, sternum, clavicle, and anterior deltoid, which raised suspicion of possible bone and organ damage. Reconstruction of chest wall defects is a challenge for surgeons due to its complex pathology and various surgical techniques. The integrity and stability of the chest well are vital to protecting the intrathoracic organs and adequate respiratory and cardiac function. When choosing the reconstructive technique, it is imperative to understand the anatomic and physiologic structures that are related to the defect [6]. The surgeon must consider the coverage needed to heal a wound and the potential alteration to respiratory mechanics created by thoracic wounds. A large majority of chest wall defects arise as a result of traumatic injuries, tumors, or infections [6]. The primary goals of reconstruction should be stabilization of the thoracic muscular and skeletal defects, obliteration of the intrathoracic dead space that may contribute to sepsis, protection of vital intrathoracic structures, and soft tissue coverage of the defects external to the thoracic cage [6].

Undergoing multiple rounds of wound debridement and washout due to persistent contamination and necrotic tissue along with IV Cefazolin promoted healing and reduced infection risk. With an open wound exposing muscle, the infection rate is high. Therefore, prompt management with antibiotics, pain medications, and surgical intervention was essential. The retrospective study of indications for chest wall reconstruction by Nute et al. evaluated various factors such as symptoms, pain, hospital stay, and outcomes. It showed that complications occurred in 80% of patients. Complications ranged from superficial wound dehiscence and seroma to deep infection requiring further debridement. Although the complication rate was high, with the appropriate care, 93% of patients underwent successful wound closure with favorable outcomes [2].

Postoperative and rehabilitative care are vital in achieving positive outcomes after surgical reconstruction. Prompt management of chest trauma with timely and optimal pain management and chest physical therapy resulted in a good outcome in the majority of polytrauma patients [4]. During the procedure, a subcutaneous drain is placed in the donor site and will remain postoperatively until the drain output is minimal and appears serious. In addition, patients are instructed to limit the range of motion exercise to allow for proper healing and minimal movement of the harvested muscle and soft tissue [6]. In this case, the patient worked closely with occupational therapy to ensure a smooth transition from postoperative inpatient care to outpatient care. The patient did not face any preoperative, intraoperative, or postoperative complications. The patient recovered well with a combined team effort and was discharged on the 17th postoperative day. The patient followed all postoperative plans and has full functionality of the thoracic muscles and left upper extremity.

## Conclusions

In this case, the patient was treated for a chest wall and shoulder trauma with third-degree burns secondary to a blast injury, a rare diagnosis that calls for a unique treatment plan. Reconstruction of chest wall defects can be a formidable challenge, and this case illustrates the importance of a multidisciplinary effort that consists of perioperative and surgical management. The treatment in this case, which involved debridement, flap surgery, and perioperative care, highlights the need for coordinated care to promote wound healing, prevent infection, and preserve function. Clinicians and surgeons should appreciate anatomy and physiology to navigate complex reconstructions effectively by choosing the appropriate reconstructive material. While this case utilized a fasciocutaneous flap, alternative approaches, such as primary closure, skin grafts, or conservative management, may be suitable depending on the wound’s complexity, muscle exposure, and burn severity. With the evolution and innovation in surgical techniques, the outcomes for chest wall reconstruction will continue to improve and allow optimal patient recovery.

## References

[1] Chrysou K, Halat G, Hoksch B, Schmid RA, Kocher GJ (2017). Lessons from a large trauma center: impact of blunt chest trauma in polytrauma patients-still a relevant problem?. Scand J Trauma Resusc Emerg Med.

[2] Aärimaa V, Rantanen J, Heikkilä J, Helttula I, Orava S (2004). Rupture of the pectoralis major muscle. Am J Sports Med.

[3] Clemens MW, Evans KK, Mardini S, Arnold PG (2011). Introduction to chest wall reconstruction: anatomy and physiology of the chest and indications for chest wall reconstruction. Semin Plast Surg.

[4] Seder CW, Rocco G (2016). Chest wall reconstruction after extended resection. J Thorac Dis.

[5] Sanna S, Brandolini J, Pardolesi A, Argnani D, Mengozzi M, Dell'Amore A, Solli P (2017). Materials and techniques in chest wall reconstruction: a review. J Vis Surg.

[6] Bakri K, Mardini S, Evans KK, Carlsen BT, Arnold PG (2011). Workhorse flaps in chest wall reconstruction: the pectoralis major, latissimus dorsi, and rectus abdominis flaps. Semin Plast Surg.

[7] Nute DW, Kusnezov N, Dunn JC, Waterman BR (2017). Return to function, complication, and reoperation rates following primary pectoralis major tendon repair in military service members. J Bone Joint Surg Am.

[8] Sah B, Pant A, Jaiswal LS, Gupta RK (2023). Penetrating chest trauma with right atrium rupture - a case report. Trauma Case Rep.

